# *Astragalus* *membranaceus* Extract Activates Immune Response in Macrophages via Heparanase

**DOI:** 10.3390/molecules17067232

**Published:** 2012-06-13

**Authors:** Qiaojing Qin, Jianying Niu, Zhaoxia Wang, Wangjie Xu, Zhongdong Qiao, Yong Gu

**Affiliations:** 1 Department of Nephrology, Shanghai Fifth People’s Hospital, Fudan University, Shanghai 200240, China; Email: qinqiaojing@sohu.com (Q.Q.); njyphd2008@yahoo.com.cn (J.N.); 2 School of Life Science and Biotechnology, Shanghai Jiaotong University, Shanghai 200240, China; Email: zhaoxiaw@sjtu.edu.cn (Z.W.); hover_xwj@sjtu.edu.cn (W.X.); zdqiao@sjtu.edu.cn (Z.Q.); 3 Department of Nephrology, Huashan Hospital, Fudan University, Shanghai 200240, China

**Keywords:** *Astragalus membranaceus* extract, macrophage, heparanase, migration, immune response mediator, immune response

## Abstract

*Astragalus membranaceus* (AM), a traditional Chinese medicinal herb, has immunoregulatory properties in many diseases. We investigated the effects and mechanism of *Astragalus membranaceus* extract (AME) in the macrophage migration and immune response mediator release. The viability of Ana-1 macrophages treated with AME was evaluated by the MTT method. The secretion and mRNA levels of IL-1β and TNF-α were measured by ELISA and RT-PCR, respectively. Macrophage migration was assayed by transwell assay. The activity of heparanase (HPA) was determined by a heparin-degrading enzyme assay. Our results didn’t show any toxicity of AME in macrophages. AME increased the activity of HPA, cell migration, mRNA levels and secretion of IL-1β and TNF-α in macrophages. Pretreatment with anti-HPA antibody reduced cell migration, secretion of IL-1β and TNF-α did not change the mRNA levels of IL-1β and TNF-α significantly in AME-treated macrophages. This suggests that AME may increase the release of immune response mediator and cell migration via HPA to activate immune response in macrophages.

## 1. Introduction

*Astragalus membranaceus* (AM), a herbal remedy in Traditional Chinese Medicine, possesses the immunoregulatory properties [1,2]. Evidence indicates that AM can activate macrophage by increasing the levels of cytokines, including TNF-α, and GM-CSF, without cytotoxic effects to modulate the immune response [3]. The innate immune response during bacterial infection is accompanied by an intense migration of macrophages at the site of infection. A verification of effects, mechanism and relationship between immune response mediators and migration in macrophages is necessary for the acceptance of AM supplements.

HPA, an endo-β-glucuronidase that cleaves heparan sulfate (HS) at specific intra-chain sites, is strongly implicated in dissemination of metastatic tumor cells [4]. When mouse macrophages were stimulated with lipopolysaccharide in the absence or presence of active HPA, HPA strongly sensitized macrophages activated by LPS *in vitro*, as indicated by a marked increase in TNF-a, IL-6, and IL-12 p35 [5,6]. Furthermore, induction of HPA in several inflammatory conditions was reported, associated with degradation of HS, remodeling of the extracellular matrix (ECM), facilitating the inflammatory cell migration towards the injury sites and releasing of chemokines anchored within the ECM network and cell surfaces [7].

Based on these previous reports, we speculated that HPA may be a key regulator of migration and immune response mediator in macrophages, so we examined the role of HPA in the AME-induced migration and expression of some immune response mediators, TNF-α and IL-1β, to evaluate the immunoregulatory effects and mechanism of AME in macrophages.

## 2. Results and Discussion

### 2.1. Effects of AME on Cell Viability

We tested the cytotoxicity of AME in macrophages by the MTT assay. Ana-1 macrophages were exposed to AME at 10, 20 and 40 μL/mL for 24 h.

**Figure 1:**
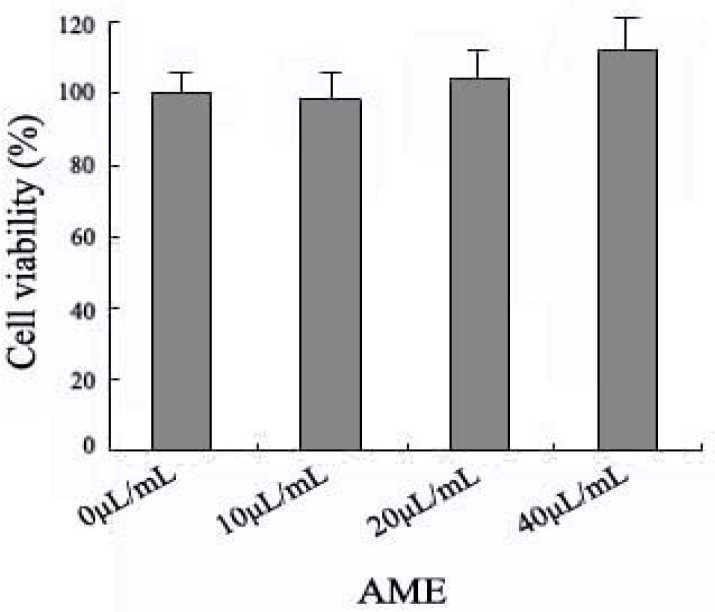
Viability analysis of AME on Ana-1 macrophages. Cells (5 × 10^4^) were treated with AME (10–40 μL/mL) respectively for 24 h and viability assay performed using MTT assay.

An increased tendency of viability was observed and 40 μL/mL AME at 24 h was the max concentration that didn’t significantly change microphage viability in the studied concentrations (Figure 1). It shows that AME don’t lead to the macrophage toxicity in the studied concentrations (10–40 μL/mL).

### 2.2. Effects of AME on HPA Enzymatic Activity

AM has been reported to possess a wide range of pharmacological properties, which include immunostimulant and anti-bacterial effects [8,9,10]. Macrophages play an important role in the regulation of the immune response involving macrophage migration and release of immune response mediators, such as IL-1β and TNF-α. HPA, an endo-β-D-glucuronidase, specifically cleaves HS and profoundly affects a variety of physiological and pathological processes including inflammation and migration of endothelial and tumor cells [11,12].

Though the immunoregulatory effects of AM have been demonstrated in many diseases, it is yet unclear how AM acts on macrophages to regulate the immune response. It is difficult to standardize the individual variations among human subjects with respect to macrophage functions, so we used Ana-1 macrophage to study the role of HPA in AME-induced migration and release of immune response mediators, IL-1β and TNF-α.

**Figure 2:**
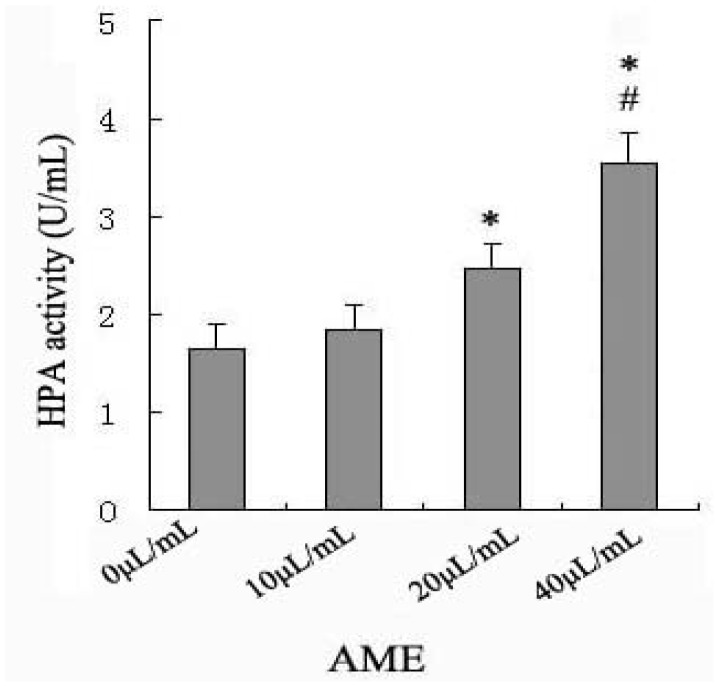
Effects of AME on the activities of HPA in Ana-1 macrophages. Cells (5 × 10^5^) were incubated with AME (10–40 μL/mL) respectively for 24 h at 37 °C. HPA activity was performed by using heparan degrading enzyme assay. * *p* < 0.05 significantly different from control (0 mg/L) and AME at 10 μL/mL; ^#^*p* < 0.05 significantly different from AME at 20 μL/mL.

We assessed the effect of AME on HPA enzyme activity in macrophages. Our results show that among the studied concentrations (10–40 μL/mL), 20 and 40 μL/mL AME increased HPA activity significantly at 24 h, with a maximal effect at 40 μL/mL (Figure 2).

To our knowledge, this is the first report that AME can activate HPA in macrophages. We speculated that AME perhaps may improve the immune response as a stimulator through activation of HPA enzyme activity in macrophages, so we chose AME 40 μL/mL at 24 h to finish the subsequent experiments associating with the mechanism.

### 2.3. Effects of Anti-HPA Antibody on AME-Induced Secretion and mRNA levels of IL-1β and TNF-α in Macrophages

HPA cleaves HS and yield HS fragments. HS sequesters a multitude of polypeptides that reside in the ECM as a reservoir. A variety of growth factors, cytokines and chemokines can be released by the activation of HPA to profoundly affect cell and tissue function [13]. We evaluated the role of HPA in the immune response mediator release from the macrophages treated with AME.

**Figure 3:**
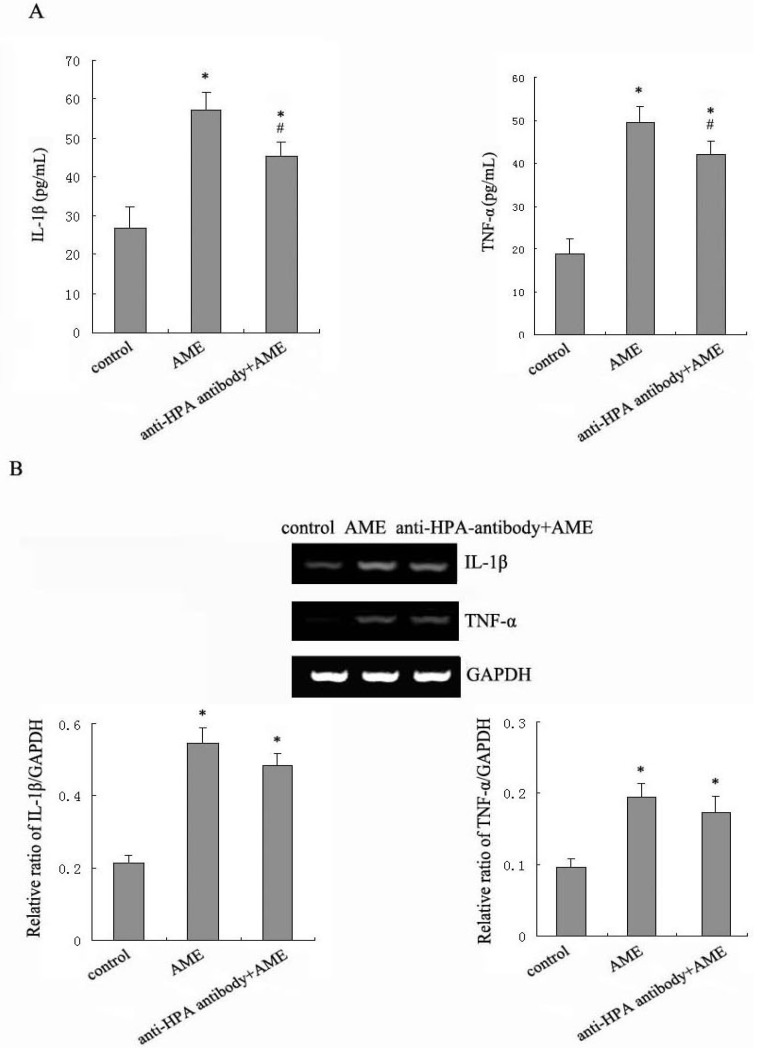
Effects of anti-HPA antibody on the expression of IL-1β and TNF-α in AME-treated macrophages. Cells were cultured with AME, pre-treated with anti-HPA antibody for 1 h before exposed to AME for 24 h. The secretion of IL-1β and TNF-α were measured by using ELISA (**A**); The mRNA levels of IL-1β and TNF-α were measured by RT-PCR (**B**). * *p* < 0.05 compared to control and ^#^*p* < 0.05 compared to AME.

In Ana-1 macrophages, the amounts of IL-1β and TNF-α were 26.82 and 18.93 pg/mL, respectively. In Ana-1 macrophages treated with AME for 24 h, the amounts of IL-1β and TNF-α increased to 56.96 and 49.51 pg/mL. Anti-HPA antibody attenuated the AME-induced production of IL-1β and TNF-α significantly to 45.22 and 42.09 pg/mL (Figure 3A).

The mRNA levels of IL-1β and TNF-α showed a pronounced increase at 24 h in the macrophages treated with AME. Pretreatment with anti-HPA antibody didn’t change the mRNA levels of IL-1β and TNF-α significantly in AME-stimulated macrophages (Figure 3B).

Our results showed that AME could significantly increase the mRNA levels and release of IL-1β and TNF-α in macrophages. Inhibition of HPA could decrease the release of IL-1β and TNF-α significantly, but not change the mRNA levels of IL-1β and TNF-α significantly in AME-treated macrophages. This suggests that the activation of HPA may be one of the pathways increasing the release of TNF-α and IL-1β to improve the immune response in AME-treated macrophages. We speculate that the effects of HPA on the release of immune response mediator may involve HS degradation or post-transcriptional regulation in macrophages. This needs further study.

### 2.4. Effects of Anti-HPA Antibody on Macrophage Migration in AME-Induced Macrophages

HPA specifically degrades the HS chains of heparan sulfate proteoglycans (HSPG) which bind to and assemble ECM proteins and plays an important role in ECM integrity, barrier function and cell-ECM interactions [14]. Enzymatic degradation of HS leads to disassembly of the ECM and is therefore involved the fundamental biological phenomena associated with tissue remodeling and cell migration [15]. Evidences have shown that the over-expression of HPA in tumor cells confers an invasive phenotype [16,17].

**Figure 4:**
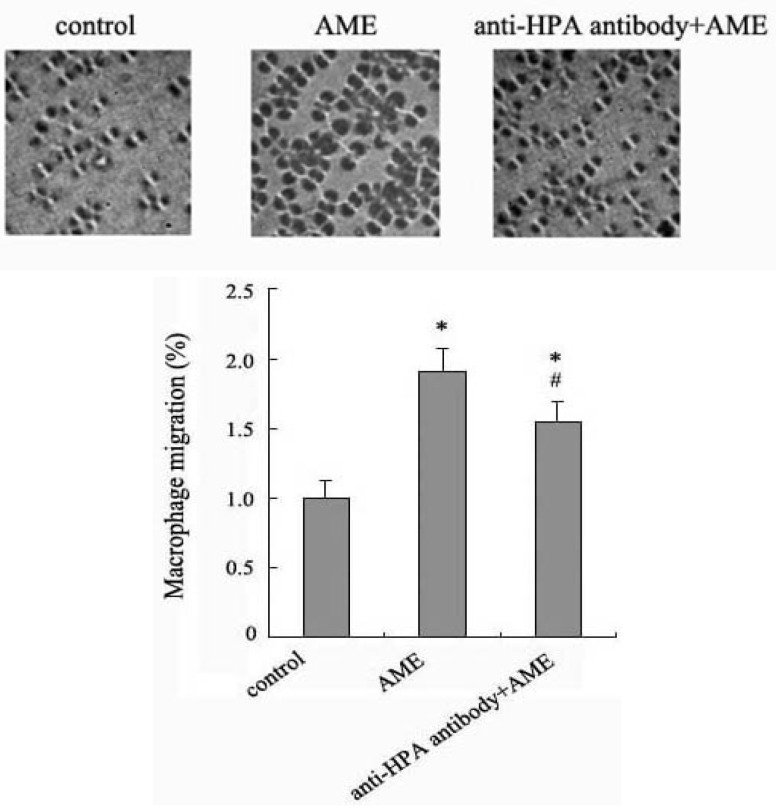
Effect of anti-HPA antibody on the migration in AME-induced macrophages. Cells were cultured with AME, pre-treated with anti-HPA antibody for 1 h before exposed to AME. The migration was measured by transwell assays. Results were normalized to the number of macrophages that migrated in control group. * *p* < 0.05 compared to control and ^#^*p* < 0.05 compared to AME.

Thus, using anti-HPA antibodies we examined the effect and mechanism of AME associated with the macrophage migration by transwell assay. Our results showed that the migration of macrophages was significantly increased after incubation with AME for 24 h. Pretreatment with anti-HPA antibody significantly prevented the upregulation of migration in AME-stimulated macrophages (Figure 4). The results suggest that AME may enhance macrophage migration via HPA to make macrophage assemble in infectious tissue and improve immune response.

## 3. Experimental

### 3.1. Materials

AME prepared according to the standard of the Chinese Pharmacopeia was from Chiatai Qingchunbao Pharmaceutical Co. Ltd. (Hangzhou, China). RPMI 1640 and fetal bovine serum (FBS) were from GibcoTM Invitrogen Corporation (Grand Island, NY, USA). RevertAid First Strand cDNA Synthesis Kit was from fermentas International Inc (Graiciuno, Vilnius, Lithuania). RNA PCR Kit was from Takara Biotechnology (DALIAN) Co. Ltd. (Dalian, China). Heparan degrading enzyme assay kit was from Takara Bio Inc. Antibody against HPA, ELISA kits for mouse TNF-α and IL-1β were from Wuhan Boster Bio-engineering Limited Company (Wuhan, China). MTT was from Sigma-Aldrich (Shanghai, China). 8.0 μm pore size transwell cell culture inserts were from Becton Dickinson and company (Franklin Lakes, NJ, USA). All other chemicals were of AR grade.

### 3.2. Preparation of AME

In brief, the dried roots of AM (1 kg), Radix AM (Fisch.) Bge var. mongolicus Hsiao, were extracted two times using ethanol (63% and 86% final concentration respectively) at 4 °C for 12 h. Then ethanol was removed by vacuum distillation. The prepared total AM was dissolved in deionized water at a ratio of 1 mL:2.5 g and filtered. The filtratrate was boiled for 5 min and deionized water added to 1,000 mL. The extract of AM was filtered and pasteurized.

### 3.3. Cell Culture

Ana-1 murine macrophage cell line was obtained from the cell bank of the Shanghai Institutes for Biological Sciences, Chinese Academy of Sciences (Shanghai, China). Cells were maintained in RPMI 1640 medium supplemented with 10% fetal bovine serum, 100 units/mL penicillin and 100 μg/mL streptomycin and were incubated at 37 °C in 5% CO_2_ humidified air.

### 3.4. Assessment of Cell Viability

Macrophages (100 μL) were seeded at a density of 5 × 10^4^ cells/mL and incubated with AME (10, 20, 40 μL/mL, respectively) in 96-well plates. After 24 h incubation, MTT solution was added to each well for 4 h. Finally, the blue salt in each well was dissolved and the plates were read by using a microplate reader with RPMI 1640 as blank and cell culture medium as control.

### 3.5. Assessment of HPA Activity

Macrophages (1 mL) were seeded at a density of 5 × 10^5^ cells/mL in 6 well plates overnight. Macrophages were cultured separately with AME at 10, 20, 40 μL/mL for 24 h. The activities of HPA were measured using the Heparan Degrading Enzyme Assay Kit (TaKaRa). This assay is based on the principle of detecting the amount of HS substrate remaining after digestion of enzyme substrate reaction. We determined the AME at 40 μL/mL to continue the following experiments according to the results of viability and HPA activity in AME-induced macrophages (the choice of dosage is explained in the discussion above).

### 3.6. Assessment of TNF-α and IL-1β Release

The Ana-1 macrophages were plated at 5 × 10^5^ cells/well in 24 well plates overnight. Macrophages were cultured separately with AME, and anti-HPA antibody (pre-treated with 10 μL/mL anti-HPA antibody for 1 h before exposed to AME) for 24 h. The levels of TNF-α and IL-1β in culture supernatants were determined using commercially available enzyme linked immunosorbent assay (ELISA) kits according to the manufacturer’s instructions.

### 3.7. Detection of mRNAs by RT-PCR

1 × 10^6^ Ana-1 macrophages were plated in a 6-well culture plate in culture medium overnight, separately with AME, and anti-HPA antibody (pre-treated with 10 μg/mL anti-HPA antibody for 1 h before exposed to AME) for 24 h. Equal amounts of RNA isolated using TRZOL Reagent from cells were reverse transcribed into cDNA using RevertAid First Strand cDNA Synthesis Kit according to the protocol by the manufacturer. Products were amplified from cDNA templates over cycled 35 times using primers for TNF-α and IL-1β (TNF-α forward: AAATTCGAGTGACAAGCCTGTAG, resverse: GAGAACCTGGGAGTAGACAAGGT, IL-1β forward: CAAGTGTCTGAAGCAGCTATGG, resverse: GAGATTTGAAGCTGGATGCTCT).The mount of TNF-α and IL-1β was determined and normalized by the amount of GAPDH cDNA.

### 3.8. Migration Assay

Macrophages used for seeding transwell inserts were harvested from the plate by incubating with AME, and anti-HPA antibody (pre-treated with 10 μg/mL anti-HPA antibody for 1 h before exposed to AME) for 24 h. 2 × 10^5^ Cells were plated into transwell inserts with 8 μm pores, which were coated with a thin layer of Matrigel and placed in a 6-well plate. 2% FBS were added to the lower chamber. Plates were incubated for 2 h at 37 °C. Cells remaining on the upper membrane surface were removed with a cotton swab. Upper wells were placed into 4% paraformaldehyde for 15 min to fix cells adherent to the underside of the membrane. Migrated cells were stained with hematoxylin and counted (6 random fields per slide) in ten 40× fields. Results were normalized to the number of macrophages that migrated in control group.

### 3.9. Statistical Analysis

Results are taken from a typical experiment and show means ± SEM of six replicates. Statistical analysis was performed by SPSS software using one-way analysis of variance (ANOVA). A value of *p* < 0.05 was considered statistically significant.

## 4. Conclusions

In summary, AME can activate macrophages to increase the migration and release of immune response mediators via HPA. AME may be an effective drug for improving host defenses to avoid infections. Further studies are required to confirm the additional effects and mechanism of HPA on the immune response in AME-stimulated macrophages.
